# Interventions associated with brown adipose tissue activation and the impact on energy expenditure and weight loss: A systematic review

**DOI:** 10.3389/fendo.2022.1037458

**Published:** 2022-12-09

**Authors:** Luis C. Perez, Laura T. Perez, Yash Nene, Guillermo E. Umpierrez, Georgia M. Davis, Francisco J. Pasquel

**Affiliations:** ^1^ Ponce Health Sciences University School of Medicine, Ponce, PR, United States; ^2^ Neurology Residency Program, SUNY Upstate Medical University, Syracuse, NY, United States; ^3^ Department of Endocrinology, Emory University School of Medicine, Atlanta, GA, United States

**Keywords:** brown adipose tissue, beta agonist, cold exposure, sildenafil, capsinoids, browning of white adipose tissue, energy expenditure and brown adipose tissue, weight loss and brown adipose tissue

## Abstract

**Background:**

Brown adipose tissue (BAT) plays a role in modulating energy expenditure. People with obesity have been shown to have reduced activation of BAT. Agents such as β-agonists, capsinoids, thyroid hormone, sildenafil, caffeine, or cold exposure may lead to activation of BAT in humans, potentially modulating metabolism to promote weight loss.

**Methods:**

We systematically searched electronic databases for clinical trials testing the effect of these agents and cold exposure on energy expenditure/thermogenesis and the extent to which they may impact weight loss in adults.

**Results:**

A total of 695 studies from PubMed, Web of Science, and Medline electronic databases were identified. After the removal of duplicates and further evaluation, 47 clinical trials were analyzed. We observed significant heterogeneity in the duration of interventions and the metrics utilized to estimate thermogenesis/energy expenditure. Changes observed in energy expenditure do not correlate with major weight changes with different interventions commonly known to stimulate thermogenesis. Even though cold exposure appears to consistently activate BAT and induce thermogenesis, studies are small, and it appears to be an unlikely sustainable therapy to combat obesity. Most studies were small and potential risks associated with known side effects of some agents such as β-agonists (tachycardia), sibutramine (hypertension, tachycardia), thyroid hormone (arrhythmias) cannot be fully evaluated from these small trials.

**Conclusion:**

Though the impact of BAT activation and associated increases in energy expenditure on clinically meaningful weight loss is a topic of great interest, further data is needed to determine long-term feasibility and efficacy.

## Introduction

Excess body weight is one of the greatest risk factors contributing to the burden of disease worldwide, leading to a decrease in life expectancy through the development of cardiovascular disease, type 2 diabetes, and cancer, among others (1). Energy balance involves an interplay between energy intake, energy expenditure, and energy storage. When energy expenditure exceeds energy intake, energy balance is negative and leads to weight loss (2). Total daily energy expenditure (EE), determined by body size, body composition, food intake, and physical activity, can be measured by using indirect, direct, or non-calorimetric methods (3). At present, the gold standard for the measurement of EE is indirect calorimetry (3). In comparison to the direct calorimetry, the method is more affordable, and presents the advantage of providing information on the metabolic fuels being combusted in addition to measuring the metabolic rate (4).

It is well established that brown adipose tissue (BAT) plays a role in modulating EE. BAT protects the neonatal body temperature at birth by producing energy in the form of heat (thermogenesis) in the mitochondria (5, 6). Thermogenesis occurs *via* the presence of uncoupling protein-1 (UCP-1) in the inner mitochondrial membrane, which transports protons across the membrane and dissipates the proton gradient formed during oxidative phosphorylation. This results in heat, instead of ATP, as the metabolic end product (6, 7). This heat dissipation can be measured *via* indirect calorimetry (3). With the recent use of PET/CT analysis, BAT was re-discovered (8) in the adult population, and is now known to be present throughout the greater part of life and involved in body weight regulation (6). Additional clinical data has shown a reduced amount of metabolically active BAT in obese subjects (9, 10). Experiments indicate that under the influence of catecholamines, thyroid hormone, capsinoids, and cold exposure, oxidative phosphorylation is uncoupled in the mitochondria of BAT, resulting in subsequent EE (11).

We systematically reviewed clinical trials examining the effects of interventions associated with changes in EE, BAT activation and associated effects on weight loss, when data was available.

## Methods

### Search strategy

We systematically searched the PubMed, Web of Science, and Medline electronic databases for clinical trials published between 2000 and 2022. We used a combination of terms including: (weight loss “OR” energy expenditure “OR” thermogenesis) “AND” (thyroid hormone “OR” cold exposure “OR” beta agonist “OR” adrenergic receptor agonist “OR” beta receptor agonist “OR” capsinoids). Limiters were consistent among the three databases (Humans “AND” Clinical Trials). Research studies were initially reviewed and selected based on the content of the title and abstract. Full content was then reviewed to determine final inclusion. The search strategy was developed and carried out by LCP, LTP, and YN.

### Inclusion and exclusion criteria

Clinical trials obtained from databases were preliminarily screened by analyzing the title and abstract. Full content was reviewed to determine final inclusion. Research studies that were not clinical trials and those that failed to mention weight loss, thermogenesis, and/or energy expenditure were excluded. Full content exclusion criteria dismissed studies from inclusion in tables where energy expenditure could not be deduced (n =2), studies on animals that were not aforementioned in the abstract or title (n=4), studies where an official article was unable to be acquired (n=1), studies that used lifestyle modifications as the intervention that elicited weight loss (n=1), studies concerning a case report on an individual patient (n=1), studies measuring the rise or fall of the intervention of interest as a result of a surgical procedure being performed on the patient (n=1), studies that were mechanistic in nature (n=1), studies where the results on energy expenditure were derived from a meta-analysis of various clinical trials (n=1), studies conducted before the year 2000 (n=9), and studies where the intervention of choice was non-pharmacological (n=5).

### Identification of relevant studies

**Figure 1:**
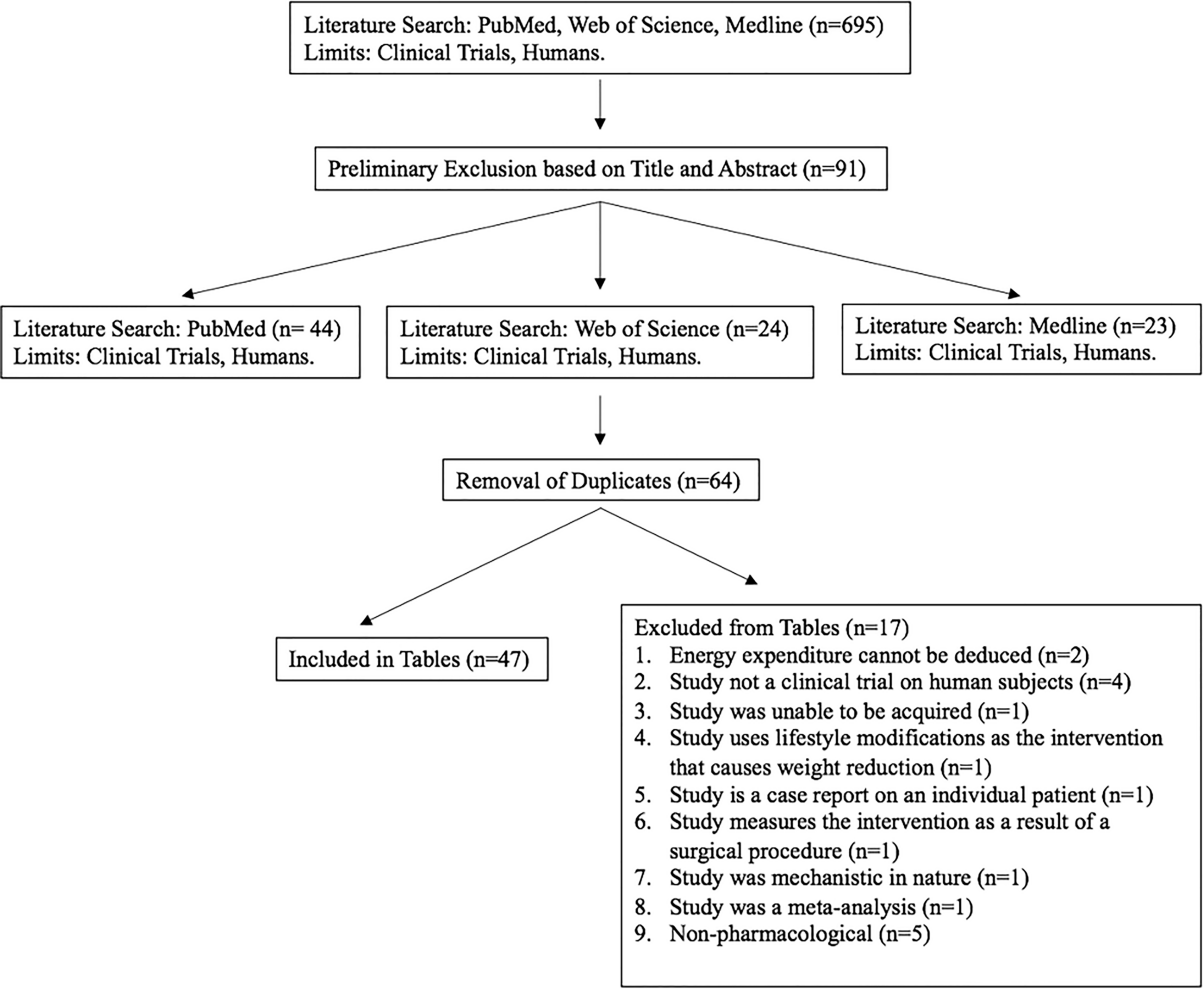
A flow chart illustrating the search, inclusion, exclusion, and results of the literature search.

A total of 695 studies from PubMed, Web of Science, and Medline electronic databases were assessed for inclusion in this review. Following preliminary exclusion, a total of 91 studies remained from those initially identified. After the removal of duplicates, 64 studies were assessed for full content exclusion and a total of 47 clinical trials were evaluated. This review includes tables for 47 clinical trials between the years 2000 and 2022 (**Figure 1**).

## Results

We generated tables for clinical trials that tested the effect of desired interventions (below) on energy expenditure and the role that BAT activation may plan on weight loss. Eleven studies tested the effect of β-agonists and two studies tested the effects of sibutramine (**Table 1**). Twenty-one studies tested the effects of cold exposure, and five studies tested the effects of capsinoids (**Supplementary Table 1**). Two studies tested the effects of thyroid hormone supplementation (**Table 2**). We also identified two studies with sildenafil (**Table 3**) and five studies with caffeine/green tea.

**Table 1:** Details of clinical trials investigating effect of β-agonists and sibutramine on energy expenditure/BAT activity.

Author	Agent	Study Design	Dose	Population	Duration	Measurements of Energy Expenditure	Effect on EE	Key Findings
Schiffelers et al. (12)	Isoproterenol (ISO) (β-3 agonist), nadolol (NAD), and propranolol (PRO) (β-1/β-2 antagonist agent).	*Randomized, single-blind, placebo-controlled trial.*	1^st^ study: 10, 20, 40 ng/kg.min ISO and then 2.5, 7.5, 15, or 40 mg NAD or PRO. 2^nd^ study: 80 mg NAD prior to ISO 50, 100, 200 ng/kg.min/saline.	8 male subjects. Age range of 19-26 yrs. Mean BMI of 22.4 kg/m^2^	10 days total. 9 visits for the 1^st^ study (1 with ISO and 8 with NAD or PRO) and 1 visit for 2^nd^ study.	EE measured by an open circuit ventilated hood calorimetry.	↑	Increasing dose of isoproterenol increased EE with prior 80 mg NAD (0.3 kg/min of EE with highest dose of 200 ng/kg.min; P< 0.05). Increase in HR with ISO. Increasing doses of NAD or PRO non-significantly decreased EE. Dose of ISO required for a 25% increase in EE averaged at 27 ng/kg.min for each subject.
Larsen et al. (13)	L 796568 (β-3).	*2-center double blind, randomized, parallel-group, placebo controlled.*	375 mg/day or placebo.	20 non-diabetic, non-smoking men age range 25-49 yrs. BMI range of 28-35 kg/m^2^	28 days	EE measured using indirect hood calorimetry.	↔	24 hr. EE for treatment and placebo groups was 92 ± 586 kJ/24 hr and 86 ± 512 kJ/24 hr respectively (P > 0.05). No major lipolytic or thermogenic effects.
Van Baak et al. (14)	L 796568, (β-3).	*2-center, 3-period, randomized, placebo- controlled crossover trial.*	250 mg, 1000 mg, placebo	12 healthy overweight to obese men. Mean age of 34.4 ± 5.8 yrs. and mean BMI of 30.7 ± 2.1. kg/m^2^	4 hours	EE measured by indirect calorimetry	↑	EE increased significantly by 8% only after highest dose of 1000 mg on obese subjects. P-value not available.
Hoeks et al. (15)	Dobutamine (DOB) (β-1) or salbutamol (SAL) (β-2), SAL + Acipimox (ACI) (lipid lowering agent).	*Randomized cross-over trial.*	4.6 mcg/kg/min in DOB group, 77 ng/kg/min in SAL group, SAL + ACI - 2 doses of 250 mg ACI given orally at time 0 and 120.	9 lean male volunteers with a mean body weight of 71.5 ± 3.2 kg and mean BMI of 22.2 ± 0.8 kg/m^2^	3 hours	EE measured by indirect calorimetry.	↑	EE increased approximately 13%. Baseline EE expenditure was 5.51 ± 0.30, 5.24 ± 0.35, and 4.97 ± 0.28 kJ/min in DOB, SAL, and SAL + ACI respectively. After 3 hr infusion, EE with DOB increased (0.58 ± 0.20 kJ/min (P< 0.05)); EE with SAL and SAL + ACI conditions increased by 0.72 ± 0.12 (P< 0.001) and 0.62 ± 0.12 (P< 0.001) kJ/min respectively.
Saraç et al. (16)	Sibutramine	*Randomized, placebo-controlled trial.*	Sibutramine 10 mg daily or placebo	60 obese women, age between 20 and 60 yrs. and with a mean BMI of 25 to 40 kg/m^2^	Subjects received Sibutramine 10 mg daily for 12 wks. or placebo	Thermogenic response was measured by using water immersion calorimetry.	↑	Decrease in BMI with placebo by 31.5 ± 2.05 kg/m^2^ to 30.4 ± 2.94 kg/m^2^ (p = 0.07). BMI decreased with treatment from 33.5 ± 4.1 kg/m^2^ to 30.9 ± 4.8 kg/m^2^ (p< 0.05). Thermogenic response in treatment changed from 1.27 ± 0.29 kcal/kg/h to 1.44 ± 0.13 kcal/kg/h and from 1.56 ± 0.27 kcal/kg/h to 1.33 ± 0.36 kcal/kg/h with placebo. Tachycardia.
Redman, et al. (17)	TAK 677 (β-3)	*Double blind, randomized, placebo-controlled trial.*	0.1 mg bid (n=21), 0.5 mg bid (n=22), or placebo bid (n=22)	65 obese men/women, mean BMI of 33.9 ± 2.1 kg/m^2^; mean age of 31.4 ± 0.9 yrs.	28 days	EE measured in metabolic chamber.	↑	Highest dose of 0.5 mg increased EE significantly compared to placebo (change from baseline, +13 ± 17 vs placebo -39 ± 18 kcal/d, P*<* 0.05). HR elevation with the 0.5 mg dose.
Rotstein et al. (18)	Sibutramine	*Randomized, double blind, placebo-controlled trial.*	Sibutramine 10 mg daily or placebo	15 obese females, age range 24-56 yrs. And BMI of >32 kg/m^2^	Test under resting, sub-max, and max conditions. Re-tested under identical conditions following 10mg/dy sibutramine for 5 days.	REE measured by an open circuit indirect calorimetry.	↔	REE for experimental group changed from 1,573.3 ± 145.3 kcal/day before to 1,622.4 ± 165.7 kcal/day after. However, most of the weight loss was achieved *via* decreased energy intake. WL was independent of EE. HR at submaximal exercise was higher. Significance unspecified.
Wijers et al. (19)	Cold and Propranolol (PRO) (antagonistic agent).	*Single blind, cross-over trial.*	160 mg PRO daily and 16°C of cold.	10 lean subjects with a mean BMI of 22.6 kg/m^2^	84 hrs. 1st 36 hrs. for baseline measurements, next 48 hrs. exposed to 16°C - once with PRO blockade and once without.	EE measured according to the Weir Equation.	↑	EE increased from 131.5 ± 1.1 W/m^2^ baseline to 134.4 ± 1.4 W/m^2^ with cold and no PRO (P< 0.05). EE increased from 125.9 ± 1.4 W/m^2^ baseline to 129.1 ± 2.3 W/m^2^ with cold and PRO (P< 0.05). PRO decreased plasma FFA levels.
Vosselman et al. (20)	Cold and Isoprenaline (ISO) (non-selective β- agonist).	*Single blind, cross-over trial.*	Increasing ISO for (6, 12, and 24 ng/kg/min) for 30 mins (doses 1 and 2) and 55 min (dose 3). 45 min in thermoneutral conditions then individualized cold for 2 hrs.	10 healthy male subjects with a mean BMI of 21.6 ± 1.6 kg/m^2^ and a mean age of 22.5 ± 2.5 yrs.	Cold experiment followed by ISO with a minimum of 1 week between both interventions. Then, 5 subjects underwent an additional ISO experiment.	EE measured using indirect calorimetry.	↑	ISO, at the highest dose of 24 ng/kg/min, EE increased by 19.7 ± 2% (P< 0.001) (1.2 kcal/min at baseline to 1.45 kcal/min with ISO). Cold exposure led to a 16% increase in EE (P< 0.001) (1.17 ± 0.1 kcal/min baseline to 1.37 ± 0.13 kcal/min with mild cold).
Lee et al. (21)	Formoterol (β-2).	*Dose finding study, step-wise incremental design with open label metabolic evaluation.*	80, 160, 320 mcg	4 subjects for first part; Mean BMI of 23 ± 2 kg/m^2^ and age of 30 ± 4 yrs. 8 for second part; mean age 31 ± 2 yrs. and BMI 24 ± 1 kg/m^2^	Weekly administration of 80, 160, and 320 mcg daily (dose finding) followed by a metabolic study with a 1 wk. treatment with 160 mcg.	EE measured by indirect calorimetry	↑	Doses of 80,160, and 320 mcg increased REE in dose finding study by 13 ± 2, 17 ± 3, and 14 ± 1%, respectively (P > 0.05). 160 mcg/day of formoterol in metabolic study increased EE by 13 ± 2% (1,500 kcal/d baseline to 1,695 kcal/d formoterol, P< 0.05). 160 mcg formoterol/day for 1 wk increased EE to 300 kcal/day, P > 0.05. Slight dose dependent increase in HR only.
Cypess et al. (22)	Mirabegron (β-3)	*Randomized, unblinded, cross-over trial.*	200 mg oral	12 healthy males with a mean age 22.2 ± 0.6 yrs. And mean BMI 22.7 ± 0.5 kg/m^2^	28 dys between first and last study. Day one: FDG-PET/CT and intervention. Then, 2 imaging days and intervention; with 48 hr. wash -out in between.	Measurement of EE not specified.	↑	Significant increase in the BMR by 203 ± 40 kcal/day (an increase of 13%, P = 0.001).
Onslev et al. (23)	Inhaled racemic formoterol (β-2)	*Randomized, double blind, placebo-controlled trial.*	2x 27 mcg, placebo.	Nineteen males, fourteen completed, BMI of 25-36 kg/m^2^ and age of 21-43 yrs.	Overnight. Rest and exercise.	EE and substrate oxidation measured by using indirect calorimetry.	↑	Increase in EE and fat oxidation. At rest, EE and fat oxidation were 12% (p< 0.001) and 38% (p = 0.006) higher for rac-formoterol than placebo. EE during rest was higher with rac-formoterol than placebo (1.55 kcal/min^-1^ vs 1.50 kcal/min^-1^; P< 0.001). P > 0.05 with exercise. Slight HR elevation.
Jessen et al. (24)	Terbutaline (β-2).	*Randomized, double blind, placebo-controlled trial.*	8x 0.5 mg daily or placebo.	67 healthy young men of 18-36 years of age, BMI of 14-22 kg/m^2^	4 weeks without concurrent training (n=23), with resistance training (n=23) or endurance training (n=21); training 3x/wk.	EE measured by indirect hood calorimetry.	↔	No change in BMR. Significantly increased lean body mass by 1.03 kg (p< 0.05) and 1.04 kg (p< 0.05) compared to placebo in the habitual and resistance training group, respectively. No effect compared to placebo in the endurance training group (p > 0.05). No tachycardia.
Baskin et al. (25)	Mirabegron (β-3)	*Randomized and placebo-controlled trial.*	200 mg dose of mirabegron.	12 healthy, lean men between the ages of 18-35.	A screening and up to 4 study visits, each separated by ≥48 hours and completed withing an 8-week window.	Measurement of EE not specified.	↑	Increased BAT activity in lean subjects. 5.8% increase in REE (4.5 kcal/hour).
Finlin et al. (26)	Mirabegron (β-3) and cold exposure.	*Randomized controlled trial.*	50 mg/day of mirabegron for 1o weeks.	32 lean and obese subjects.	Exposure to cold (30-minute ice pack application each day for 10 days of the upper thigh) or treated with mirabegron.	Measurement of EE not specified.	↑	Increase in WAT UCP1 activity was 1.5-fold in both lean and obese, greater than in the cold intervention group.
Nahon et al. (27)	Mirabegron (β-3) and cold exposure.	*Randomized, double-blind, placebo controlled cross-over study.*	200 mg dose of mirabegron.	10 lean Dutch south Asian male subjects and 10 lean Europid male subjects.	Three interventions consisting of 2-hour cold exposure, mirabegron, and placebo.	EE measured by indirect calorimetry.	↑	Increased armpit and supraclavicular skin temp. in Europids (+10 C, P<0.1 and +1.6 C, P<0.001 respectively) and South Asians (+0.8 C, P<0.5 and +1.7 C, P<0.001 respectively). Increased armpit and supraclavicular skin temp. in Europids (+0.6 C, P<0.05 and +0.4 C, P<0.05 respectively) and South Asians (+0.03 C, P<0.01 and +0.7 C, P< 0.01 respectively). Increased FFA levels in Europids and South Asians (+214%, P<0.001 and +155%, P<0.001 respectively). Cold exposure increased REE in both Europids and South Asians (+20%, P<0.01 and +29%, P<0.05).
O’Mara et al. (28)	Mirabegron (β-3)	*Open label study.*	100 mg dose of mirabegron.	14 healthy women between the ages of 18 and 40.	4-week treatment.	EE measured by indirect calorimetry.	↑	Increased BAT metabolic activity (195 to 473 mL.g/mL, P = 0.039) and increased BAT volume (72 to 149 mL, P = 0.036) as measured by (^18^F-FDG) PET/CT. Increase in the REE by 10.7% (6.4 kcal/h, P< 0.001) with the initial dose of mirabegron on day 1, but not with day 28 dose. However, the baseline REE on day 28 of 5.8% was higher compared to the REE prior to the drug exposure on day 1 (+82 kcal/d, P = 0.01).

EE, energy expenditure; REE, resting energy expenditure; GIT, glucose-induced thermogenesis; CIT, cold-induced thermogenesis; FFA, free fatty acid; SFT, skin fold thickness; BAT, brown adipose tissue; RMR, resting metabolic rate; HR, heart rate; TAG, triacylglycerol; UCP, uncoupling protein.

**Table 2:** Details of clinical trials investigating effect of Thyroid Axis on energy expenditure/BAT activity.

Authors	Agent	Study Design	Dose	Population	Duration	Measurement of EE/BAT Activity	EffectEE/BAT	Key Findings
Broeders, E. P. M. et al. (29)	Levothyroxine (synthetic thyroid hormone (T4)) and cold exposure.	*Longitudinal study.*	137.75 ± 23.75 µg/day	10 patients (8 females/2 males) with well-differentiated thyroid carcinoma eligible for surgical treatment and radioactive iodine ablation therapy.	2 conditions: 4-6 months with administration of Levothyroxine (subclinical hyperthyroid state) and measurement 6-8 weeks after surgical removal of the thyroid gland (hypothyroid state).	EE measured by indirect calorimetry. BAT activity measured using FDG-PET/CT analysis.	↑/↑	Significant increase in RMR after treatment in the hyperthyroid state (BMR: 3.8 ± 0.5 kJ/min vs. 4.4 ± 0.6 kJ/min, p = 0.012). NST increased from 15 ± 10% to 25 ± 6% (p = 0.009). Mean BAT activity increased in the subclinical hyperthyroid state (SUV of 4.0 ± 2.9 vs. 2.4 ± 1.8, p = 0.039). Mean Tsk significantly lower in hypothyroid state. High levels of thyroid hormone are associated with a higher level of cold activated BAT.
Heinen, C. A. et al. (30)	TRH or placebo and cold exposure.	*Randomized, double-blind, placebo controlled, cross-over.*	400µg TRH or 2 mL saline and Mild cold exposure (17°C ± 1°C), or placebo.	16 healthy lean men.	2 scans: 1-3 weeks apart.	BAT activity was measured as standardized FDG uptake and glucose metabolic rate (MRglu) measured using dynamic PET/CT imaging. Measurement of EE not specified.	↔/↔	Experiment #1 at room temp: no significant changes in BAT activity. Experiment #2 at mild cold exposure: BAT glucose uptake visibly higher in 4/9 subjects after TRH compared to placebo. Overall effect is an increase in BAT glucose uptake.

EE, energy expenditure; BMR, basal metabolic rate; NST, non-shivering thermogenesis; BAT, brown adipose tissue; SUV, standard uptake value; Tsk, skin temperature; TRH.

**Table 3:** Details of clinical trials investigating effects of Sildenafil on energy expenditure/BAT activity.

Author	Agent	Study Design	Dose	Population	Duration	Measurement of energy expenditure	Effect on energy expenditure	Key findings
Li et al. (31)	Sildenafil	*Randomized, double blinded, placebo controlled, parallel group trial*	100 mg/day sildenafil in fractionated dose (25 mg at 8AM plus 25mg at 4PM plus 50mg at 10PM) VS identical placebo	16 newly diagnosed Chinese male overweight subjects, age 20-30	7 days	Measurement of EE not specified.	↑	Increase from baseline in RMR [1465 ± 196 kcal/day (pre) vs 1642 ± 166 kcal/day (post), P<0.01]. No significant changes in weight.
Zemel et al. (32)	NS-0200 (combination of leucine, metformin and sildenafil)	*Randomized, placebo controlled, double blinded, phase 2 multicenter study*	Placebo, Low dose NS-0200 (1.1g leucine/0.5g metformin/0.5mg sildenafil) and high dose NS-0200 (1.1g leucine/0.5g metformin/1mg sildenafil)	91 subjects, age 18-75, BMI 25-40, stable health and body weight for the preceding 12 weeks, MRI-PDFF>/= 15%, ALT>/= 30U/L for men and 19U/L for women.	16 weeks	Measurement of EE not specified	Not specified.	NS-0200 dose responsively reduced weight by 2.4kg in the study cohort.

EE, energy expenditure; RMR, resting metabolic rate.

### Beta receptor agonists

**Figure 2:**
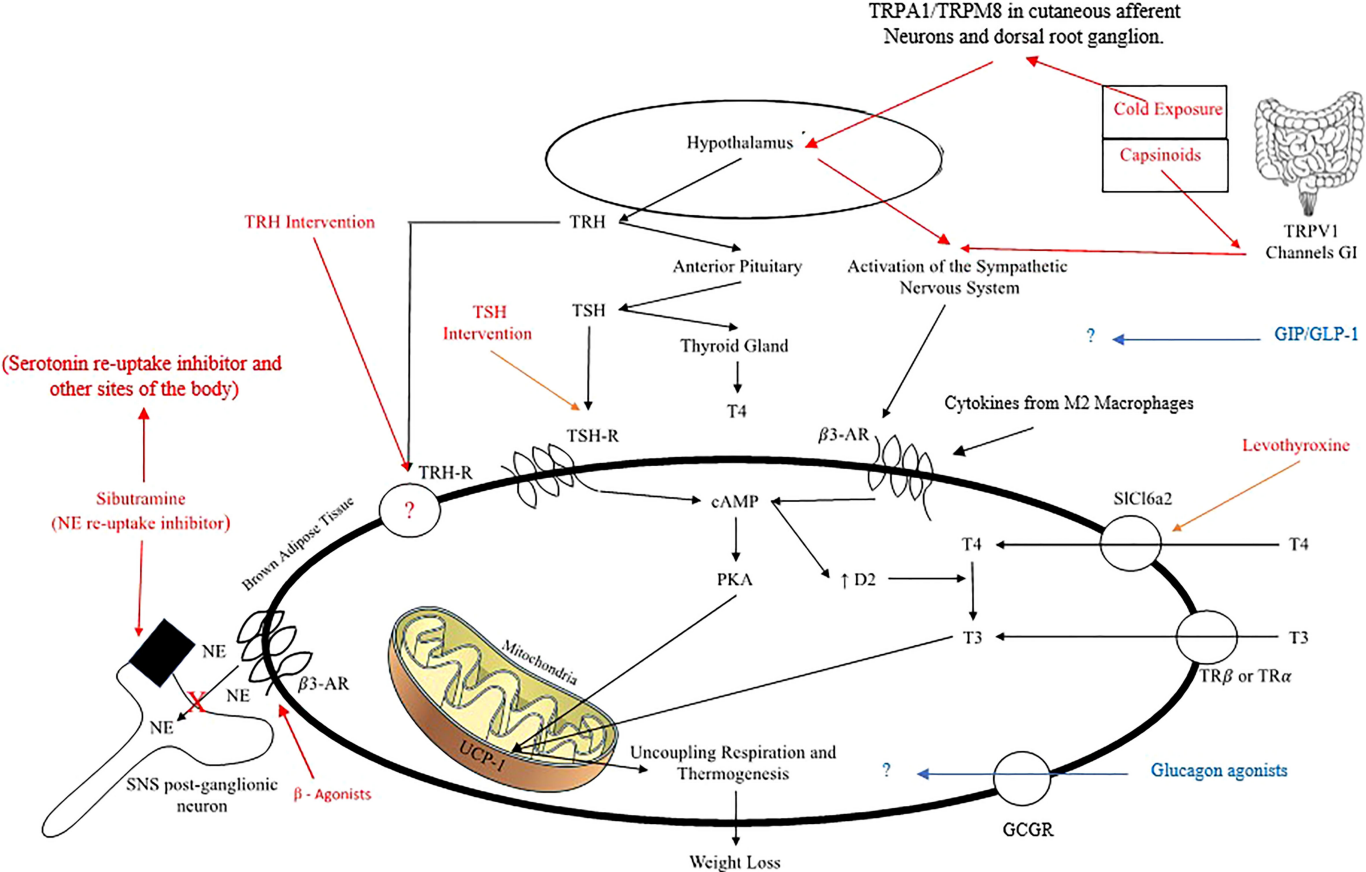
Mechanisms of pharmacological and non-pharmacological, direct and indirect activation of Brown Adipose Tissue to produce thermogenesis.

Catecholamine binding to receptors on BAT plasma membrane leads to a rise in intracellular 3’,5’-cyclic adenosine monophosphate AMP (cAMP) and subsequent activation of cAMP-dependent protein kinase A (PKA), which then phosphorylates target proteins and genes responsible for uncoupling mitochondrial respiration (**Figure 2**) (33); catecholamine binding also mediates the promotion of fat oxidation and the release of free fatty acids (FFA) that contribute to the activation of uncoupling protein 1 (UCP-1) (34).

One study tested a short-term (1 week) treatment of the non-selective β-agonist, isoprenaline (20). There was a 19.7% increase in EE on lean subjects with isoprenaline (1.2 kcal/min baseline to 1.43 kcal/min, P< 0.001) at the highest dose, an increase in proximal skin temperatures and heart rate, but only 1/10 subjects showed detectable BAT activity despite the increase in EE that was observed (20). BAT activity was measured *via* “2-deoxy-2-[(18)F]fluoro-d-glucose ([(18)F]FDG) positron emission tomography/computed tomography.

Three studies tested the use of selective β-2 agonists (21, 23, 24). Two of these studies showed a dose dependent increase in EE in subjects receiving formoterol (21, 23); one study on lean subjects showed an increase in EE by 13% with the middle dose of formoterol (1,500 kcal/day baseline to 1,695 kcal/day, P< 0.05), a lower respiratory exchange ratio (RER) and higher plasma FFA levels (21), and an additional study with formoterol on overweight subjects showed that the resting energy expenditure (REE) was higher in the treatment group than with placebo (1.55 kcal/min^-1^ vs 1.50 kcal/min^-1^, P< 0.05), with an associated increase in fat oxidation and heart rate (23). Lean subjects receiving terbutaline showed no change in the resting metabolic rate (RMR), but had an increase in the lean body mass (24).

Nine studies tested the use of selective β-3 agonists (12–14, 17, 22, 25, 26, 28, 35). Two of these studies showed a dose dependent increase in EE on overweight or obese subjects when using L 796568 and TAK 677 (13, 14, 17). An acute administration (4 hours) of L 796568 resulted in a significant increase in EE by 8% at the highest dose and an increase in plasma FFA (14). The change in EE from baseline when using TAK 677 was 13 ± 17 kcal/day at the highest dose vs -39 ± 18 kcal/day using placebo, P< 0.05, with an increase in plasma FFA levels as well (17). On the contrary, a 28 day intervention (28 days) of L 796568 resulted in no significant effect on EE (92 kJ/day vs the 86 kJ/day with placebo, P > 0.05) (13). Two studies on lean subjects showed an increase in EE when using isoproterenol (β-2) and mirabegron (β-3) (12, 22). The dose of isoproterenol required for a 25% increase in EE averaged at 27 ng/kg.min for each subject (12); however, even after pretreatment with nadolol and propranolol, the increase in EE, plasma FFA and glycerol, and the decrease in RER could not be solely attributed to the β-3 agonism of isoproterenol without evidence of some contributing β-1 (and possibly β-2) activity. Additionally, subjects receiving mirabegron showed an increase in the RMR by 203 ± 40 kcal/day (13%) (22). A newer study assessing the effects of mirabegron shows an increase in white adipose tissue (WAT) UCP1 activity of approximately 1.5-fold following 10 weeks of treatment in both lean and obese subjects as demonstrated by immunohistochemistry from thigh and abdominal adipose tissue biopsies; significantly greater than that observed with a 10-day cold exposure intervention (26). Another study assessing the effects of mirabegron on lean subjects with previously detectable cold-activated BAT showed that 200 mg of mirabegron increased BAT activity as demonstrated with PET imaging, as well as increased the REE by 5.8% (4.5 kcal/h, P = 0.02) (25). An additional study observed that a 4-week, chronic use of 100 mg of mirabegron (higher than the 50 mg approved dose) on young healthy women was associated with increased BAT metabolic activity (195 to 473 mL.g/mL, P = 0.039) and increased BAT volume (72 to 149 mL, P = 0.036) as measured by (^18^F-FDG) PET/CT; with women who had lower levels of BAT at the beginning of the study seeing larger increases. In addition, there was an increase in the REE by 10.7% (6.4 kcal/h, P< 0.001) with the initial dose of mirabegron on day 1, although the day 28 dose of mirabegron did not increase the REE any further; however, the baseline REE on day 28 of 5.8% was higher compared to the REE prior to the drug exposure on day 1 (+82 kcal/d, P = 0.01) (28).

A smaller study by Hoeks et al. tested the use of both selective β-1 and β-2 agonists on lean subjects (15); thereby showing an increase in EE by 10% with dobutamine, a β-1 agonist (5.5 kJ/min baseline to 6.08 kJ/min, P< 0.05), an increase in plasma FFA, glycerol, and heart rate, and a decreased RER. Additionally, there was an increase in EE by 13% with salbutamol, a β-2 agonist (5.24 kJ/min baseline to 5.96 kJ/min, P< 0.001), and an increased plasma FFA and glycerol (15) (**Table 1**).

In summary, 13 out of 15 studies showed an increase in EE with exposure to beta agonists. Out of these, four studies investigated beta agonists in combination with cold exposure, and all four showed an increase in EE. Nine of the studies involved beta 3 agonists, and eight of these showed an increase in EE.

### Sibutramine

Two studies tested the use of sibutramine (16, 18). Sibutramine is a serotonin/noradrenaline reuptake inhibitor which contributes to an increase in BAT activity by increasing sympathetic nervous system activity (36). The most significant concerns with sibutramine are blood pressure elevations and tachycardia; however, the drug is allowed for use on the market in small doses (**Figure 2**). Two clinical trials showed an increase in energy expenditure when using sibutramine (16, 18). One study showed statistically significant weight loss in all subjects, with a change in BMI from 33.5 ± 4.1 kg/m^2^ to 30.9 ± 4.8 kg/m^2^ after 12 weeks (P< 0.05) as well as an increase in the thermogenic response from 1.27 kcal/kg/hr to 1.44 kcal/kg/hr (16). Another study showed an increase in the REE from 1573 kcal/day to 1622 kcal/day (P< 0.05), although no significant change in EE could be attributed to sibutramine in the treatment group, and weight loss was attributed to reduced intake (18) (**Table 1**).

### Capsinoids

Six studies tested the use of capsinoids (37–42). Capsinoids activate transient receptor potential vanilloid subtype 1 (TRPV1) calcium channels in the intestines, which results in release of catecholamines, and subsequent SNS activity stimulating BAT adrenoreceptors (40, 43); thereby, indirectly activating BAT and ultimately upregulating UCP-1 (39, 40). Additionally, evidence suggests that capsinoids promote lipid oxidation and calcium entry necessary to prevent preadipocyte-to-adipocyte differentiation; therefore, TRPV1 may ultimately be used to reduce the number and size of adipose tissue (43) (**Figure 2**).

Three studies with capsinoids reported an increase in REE (37–39); with one also showing modest weight loss in overweight subjects (-0.9 kg with capsinoids vs -0.49 kg with placebo) (38).

Three studies, all conducted on lean subjects, tested the use of cold exposure compared to capsinoid ingestion (40–42). One study showed that capsinoids stimulated BAT activity to a lesser degree than cold exposure (42). The other two studies used a combination of capsinoids and cold exposure. Significant findings showed prominent FDG uptake in the supraclavicular region after 1 hour of cold followed by capsinoid ingestion with EE increasing from baseline by 15.2 kJ/day in the BAT (+) group (40), and a higher EE in subjects who consumed capsinoids after being exposed to cold (41) (**Supplementary Table 1**). In summary, all six studies investigating the effects of capsinoids on EE showed an increase in EE but modest or no effect on weight loss in studies reporting weight changes.

### Cold exposure

Cold exposure stimulates BAT activation and increases EE through stimulation of TRPA1/TRPM8 channels at cutaneous afferent neurons and dorsal root ganglia. These afferent signals reach the hypothalamus, which then acts on increasing the activity of the sympathetic nervous system (44) (**Figure 2**). Twenty-two studies tested the use of cold exposure. Fourteen studies found increases in EE using a variety of techniques (45–58). Three of these studies measured differences in EE between thermoneutral and cold conditions (46, 47, 55). Significant findings demonstrated an increase in EE from thermoneutral (obese: 12.92 mJ/day; lean: 11.3 mJ/day) to mild cold conditions (obese: 12.97 mJ/day; lean: 11.60 mJ/day) (P< 0.01) (47); an increase in EE from 1701 kcal/day at thermoneutral to 2052 kcal/day with cold in overweight subjects (P = 0.046) (55); and an increase in EE from 82 kcal/hr at thermoneutral to 86 kcal/hr at 19°C in lean subjects (P< 0.001) (46). Two studies measured differences in EE between BAT (+) and BAT (-) subjects (48, 51). BAT (+) vs BAT (-) individuals represent those who showed detectable FDG-uptake in the supra- and sub-clavicular regions after cold-stimulation and those who showed undetectable FDG-uptake, respectively (48). Significant findings demonstrated an increase in EE from 1446 kcal/day at 27°C to 1856 kcal/day at 19°C for BAT (+) lean subjects vs the increase to 1475 for BAT (-) subjects (P< 0.05) (48); another study showed an increase in the cold-induced thermogenesis (CIT) by 252 kcal/day for BAT (+) lean subjects vs the 78 kcal/day observed for BAT (-) subjects (P< 0.01) (51). Three other studies showed an increase in EE (45, 50, 56). A short-term (3 day) study by Wijers et al. showed an increase in EE by 0.59 mJ/day in lean subjects (P< 0.001) (45). Another study demonstrated an increase in the CIT from 5.2% to 12% and an increase in the RMR from 1782 kcal/day to 1824 kcal/day in lean to overweight subjects (56). An additional study showed an increase in EE in lean females (6.2 to 6.9 mJ/day) and male (7.6 to 8.5 mJ/day) subjects (P< 0.05) (50). Two studies showed a decrease in EE following cold acclimation, however, an increase in EE within the cold acclimated vs non-cold acclimated group (52, 54); an increase in EE both before (5.9 to 6.5 kJ/min) and after cold acclimation (5.7 to 6.5 kJ/min) in overweight subjects (P< 0.01) (54) and an increase in EE both before (1.4 to 2.7 kcal/min) and after cold acclimation (1.3 to 2.5 kcal/min) in lean subjects (52). Limitations to the above finding include a study demonstrating an 8.3% decrease in EE (59), and an additional study finding no effect on EE after cold stimulation (60). One short term (5 days) study identified a reduction in weight by 0.5 kg in lean subjects (P< 0.05) (56).

Five studies made a comparison of β-agonist vs cold exposure and capsinoids vs cold exposure based on their effect on EE (19, 20, 40, 41, 51). A study by Vosselman et al. (**Table 1**) compared cold exposure with the non-selective β-agonist isoprenaline on lean subjects (20), showing a CIT of 16.9% (1.17 kcal/min baseline to 1.37 kcal/min, P< 0.001) vs an increase in EE with isoprenaline by 19.7% (1.2 kcal/min baseline to 1.45 kcal/min, P< 0.001). The β-receptor antagonist propranolol was tested with cold exposure on lean subjects in one of the studies, showing an increase in EE with cold exposure only (131 W/m^2^ baseline to 134.4 W/m^2^, P< 0.05) and a lower increase in EE with the addition of propranolol to cold exposure (125.9 W/m^2^ baseline to 129.1 W/m^2^, P< 0.05) (19). One study measuring the effects of the β-3 agonist mirabegron and cold exposure on young, lean Europid and South Asian participants found that cold exposure increased armpit temperature (as a proxy of core body temperature) and supraclavicular skin temperature in Europids (+10 C, P<0.1 and +1.6 C, P<0.001 respectively) and South Asians (+0.8 C, P<0.5 and +1.7 C, P<0.001 respectively). Mirabegron also increased armpit and supraclavicular skin temperature in Europids (+0.6 C, P<0.05 and +0.4 C, P<0.05 respectively) and South Asians (+0.03 C, P<0.01 and +0.7 C, P< 0.01 respectively). Mirabegron increased FFA levels in Europids and South Asians (+214%, P<0.001 and +155%, P<0.001 respectively). Additionally, cold exposure increased REE in both Europids and South Asians (+20%, P<0.01 and +29%, P<0.05); however, no increase in REE with mirabegron use over time (27).

Cold exposure was also administered to lean subjects in combination with capsinoids (**Table 3**), showing an EE of 146 kcal/2 hrs with capsinoid treatment and a rise to 300 kcal/2 hrs with cold (42). Three studies showed an increase in EE following cold acclimation (40, 41, 51). One showing an increase in EE in BAT (+) subjects receiving capsinoids after cold acclimation (15.2 ± 2.6 kJ/d vs 1.7 ± 3.8 kJ/d BAT -) (P< 0.01) (40); another showing an increase in the CIT in lean BAT (+) subjects receiving capsinoids after cold acclimation (252 ± 41.1 kcal/d vs 78.4 ± 23.8 kcal/d BAT -) (P< 0.01) & it was found that in individuals with low or undetectable BAT the CIT after capsinoid treatment was directly proportional to BAT activity, further showing the importance of capsinoids for the activation of BAT (51); lastly, one study showed an increase in the fat oxidation and EE in the lean BAT (+) group receiving capsinoids following cold acclimation (P = 0.01) (41) (**Supplementary Table 1**). In summary, a total of 28 studies investigating the effect of cold exposure on EE (alone or in combination) were reviewed (**Table 1** and **Supplementary Table 1**). Out of these, a total of 25 studies showed an increase in EE. Most studies were of short duration and the impact on weight loss is uncertain.

### Thyroid hormone

Thyroid hormone stimulates all cells in the body and influences the basal metabolic rate under the regulation of thyrotropin-releasing hormone (TRH) from the hypothalamus and thyroid stimulating hormone (TSH) from the pituitary. Thyroid hormone in the form of thyroxine (T4) is converted to a more active form, triiodothyronine (T3) in peripheral tissues such as the liver, kidneys, and BAT; with T3 having the ability to enhance uncoupling respiration in BAT (29). Interestingly, TSH-receptors have been shown to be present on BAT plasma membrane in mice (29). This points to both TSH and T3 in having great importance in BAT function and activation of uncoupling respiration (29) (**Figure 2**). One study tested the use of TRH (30) and another the use of levothyroxine (synthetic exogenous thyroid hormone) (29). Both of these studies included cold exposure as an agent alongside thyroid hormone therapy. The study using levothyroxine as the intervention resulted in an increase in the RMR, an increase in BAT activity, an increase in EE, an increase in non-shivering thermogenesis (NST) from 15% to 20%, with lower mean skin temperatures (29). The second study involving TRH administration following mild cold exposure resulted in an increased BAT glucose uptake compared to the administration of TRH at room temperature (30) (**Table 2**).

### Sildenafil

Phosphodiesterase type 5 (PDE5) inhibitors and their effect on energy expenditure through the induction of a BAT thermogenic phenotype in white adipose tissue (WAT) mediated by cGMP, remains a topic of interest. One study investigated short-term (7 day) treatment with sildenafil in overweight Chinese men (N=16) and potential “browning” of subcutaneous WAT (31). Results showed an increase from baseline in RMR [1465 ± 196 kcal/day (pre) vs 1642 ± 166 kcal/day (post), P<0.01] with associated increases in norepinephrine [1.88 ± 0.71 nmol/L (pre) vs 2.91 ± 0.72 nmol/L (post), P<0.05], epinephrine [0.13 ± 0.03 nmol/L (pre) vs 0.17 ± 0.04 nmol/L (post), P<0.05] and cGMP [22.13 ± 9.14 pmol/mL (pre) vs 65.50 ± 13.72 pmol/mL (post), P<0.01) levels after sildenafil treatment that were not observed in the placebo group. No significant changes in weight were observed in either group. Evaluation of subcutaneous WAT in sildenafil-treated subjects revealed a smaller, more loculated morphology with evidence of increased UCP-1 expression on immunohistochemical staining and an increased density of mitochondria on electron microscopy when compared to those treated with placebo, consistent with a more thermogenic BAT phenotype (31). Further data using functional PET/CT imaging of BAT depots did not reveal in increase in activity or expansion of the pre-existing BAT tissue, confirming the hypothesis that sildenafil works not by BAT activation, but through inducing a more thermogenic (“beige”) phenotype in existing WAT (31). An additional study evaluated the effects of NS-0200 on weight loss in obese subjects over 16 weeks compared to placebo (N=71) (32). NS-0200 contained a combination of leucine, metformin and sildenafil, hypothesizing a synergistic effect between the components to increase metabolic effects mediated by AMP-activated protein kinase (AMPK) and mammalian sirtuin 1 (Sirt1) *via* direct Sirt1 activation (leucine) and indirectly through endothelial nitric oxide synthase (sildenafil). Although a significant 2.4kg weight loss was observed between high dose NS-0200 and placebo groups, the study did not directly evaluate changes in energy expenditure or changes associated with adaptive thermogenesis. Metformin is known to affect weight, and the findings are difficult to interpret (**Table 3**).

### Caffeine/green tea extract

Caffeine may promote BAT function at thermoneutrality, peroxisome proliferator-activated receptor gamma coactivator 1-alpha expression, and mitochondrial biogenesis (61). Findings from a small study by Dulloo et al. (N=10) suggested green tea extract exposure (high content in caffeine and catechin polyphenols) results in a significant increase in 24-hour EE (4%; P< 0.01) compared to placebo; however, treatment with caffeine alone (similar content) had no effect on EE (62). In a small study (N=8) examining non-shivering thermogenesis during cold exposure, EE was approximately 10% higher during green tea exposure compared with placebo (P=0.007) (63). A larger study (N=80) tested bioactive food ingredients (tyrosine, capsaicin, catechins, and caffeine) on thermogenesis, body fat loss and fecal fat excretion after a weight loss intervention. Those who lost weight were randomized to the bioactive supplement (N=57) or placebo (N=23). The bioactive supplement increased 4-hour thermogenesis by 90 kJ compared to placebo, an effect that was maintained after 8 weeks and was associated with a slight reduction in fat mass 0.9 kg (0.5; 1.3), suggesting the compound may support weight maintenance after a hypocaloric diet (64). A previous experiment (N=120) showed no effect of green tea did on body-weight maintenance after a 7.5% bodyweight loss compared with placebo in originally overweight individuals. In addition, no differences in metabolic and blood parameters were observed between the green tea and the placebo group (65). A study investigating the acute and chronic effects of oral administration of catechin with caffeine on whole-body EE and BAT activity in humans (N=15) assessed with PET/CT, showed a single ingestion of the catechin beverage increased EE in 9 subjects who had metabolically active BAT (mean ± SEM: +15.24 ± 1.48 kcal, P< 0.01) but not in 6 subjects who had minimal BAT activity (mean ± SEM: +3.42 ± 2.68 kcal). Chronic exposure also led to an increase in cold induced thermogenesis (from 92.0 ± 26.5 to 197.9 ± 27.7 kcal/d; P = 0.009) (66).

## Discussion

The ability to exploit the thermogenic potential of BAT in the adult human body to promote energy expenditure and achieve weight loss or an improved metabolic phenotype are ongoing. We identified a wide range of interventions and examined the effects of β-agonists, cold exposure, capsinoid ingestion, sibutramine, thyroid hormone, sildenafil, and caffeine on EE and the role that BAT plays in this process for effective long-term weight loss. Though promising in theory, many trialed interventions targeting various mechanisms leading to BAT activation or expansion have shown little effect on sustainable weight loss. Some findings are promising (i.e. β-3 agonists, cold exposure) but most studied approaches may be impractical, minimally effective, or associated with significant side effects.

The identification of functional cold activated BAT in adult humans stimulated the discussion of its importance in human physiology and metabolism in the past decade (67). Given that some agents, known to activate BAT, had previously examined changes in EE or changes in weight we expanded our search to the last two decades. The assessment of BAT activation in clinical trials may be challenging, however newer markers (i.e. cell membrane specific markers) are under investigation (67). A commonly used technique to assess BAT activation in humans is positron emission tomography/computed tomography (PET/CT) scan with a radioactive labelled glucose tracer. Besides substrate uptake, several other aspects of BAT activity can be measured such as oxidative metabolism, local blood perfusion, and sympathetic innervation (67). The contribution of activated BAT to whole body EE is not well known. With is limitations, thermogenesis may be commonly estimated with indirect calorimetry, which has been used historically to obtain accurate measurements of EE (68). For a true assessment of BAT activation in different trials, the methodologies identified in this review are under conditions with agents known to activate BAT.

The average rates of total daily energy expenditure (TDEE) for men and women in the United States are estimated at 2850 kcal/day (or 1.97 kcal/min) for men and 2266 kcal/day (or 1.57 kcal/min) for women (69). The β-2 agonist, formoterol, significantly increased EE by 1.55 kcal/min (p<0.05) in overweight subjects (23). The non-selective β-agonist isoprenaline increased energy expenditure by 0.23 kcal/min (p<0.001) in lean subjects, however, it did not activate BAT as measured by 18F-fluorodeoxyglucose (^18^F-FDG), suggesting that other tissues are responsible for the increased beta-adrenergic thermogenesis (20). Selective β-3 agonists are known to stimulate rodent BAT (22). The use of the β-3 agonist nebivolol in human adipocytes can induce lipolysis and promote thermogenic as well as mitochondrial gene expression (including UCP-1) (35). The use of 200 mg of oral mirabegron led to higher BAT metabolic activity as measured *via* 18F-fluorodeoxyglucose (^18^F-FDG) uptake in all subjects (p = 0.001), as well as producing an increase in the RMR by 0.14 kcal/min (+13%; p=0.001) (22). Additionally, BAT metabolic activity was also a significant predictor of the changes in the RMR (p=0.006) (22). In addition to producing an increased BAT metabolic activity and increased BAT volume (25, 28), mirabegron can also increase WAT UCP1 activity by 1.5-fold in both lean and obese individuals (26). These findings suggest the use of β-3 adrenergic receptor agonists may be promising to treat metabolic diseases. However, an increase in HR (14 ± 3 bpm) and systolic blood pressure (11 ± 2 mmHg) was observed with high-dose mirabegron (22), making this approach undesirable compared to currently approved agents to treat obesity.

The greatest production of EE in the cold exposure interventions was 0.28 kcal/min in BAT (+) subjects (p<0.05) (48). EE seems to be greater with cold intervention tested on BAT (+) subjects (48, 51). One study showed a 2% reduction in weight following cold exposure, with a measured CIT in BAT (+) subjects of 0.175 kcal/min (p<0.05) (51) Only one study produced a reduction in weight of 1.1 lbs (0.5 kg) (p>0.05) after cold exposure (56), indicating that cold exposure may not be a feasible treatment for weight reduction in obese patients. Cold exposure with the non-selective β-agonist isoprenaline showed a CIT of 0.2 kcal/min (p<0.001) (20). Cold exposure with the β-3 agonist mirabegron showed an increase in armpit and supraclavicular skin temperatures. When Capsinoid treatment was followed by cold exposure this produced a greater increase in EE (2.5 kcal/min) (42) compared to when cold exposure was followed by capsinoid treatment (40, 51). Additionally, in studies where cold exposure that was followed by capsinoid treatment, those with BAT (+) subjects showed a greater increase in EE (51). It is possible that the effect on EE can be augmented with the use of cold exposure plus capsinoid intervention, with the use of cold exposure plus β-agonist intervention, as well as with the use of cold exposure plus thyroid hormone intervention (as demonstrated by an increase in BAT glucose uptake) (30).

In studies testing capsinoids, only one produced a reduction in weight of 2 lbs (0.9 kg), indicating that capsinoid intervention on its own does not result in major changes in weight necessary for long term weight management strategies (38). On the other hand, it is well known that sibutramine promotes weight loss (70), which was seen in one study demonstrating a change in the BMI in all subjects (16), however, it remains unclear whether this weight loss is due to a sibutramine-induced increase in EE or as a result of decreased caloric intake (18).

Heart rate, shivering, and other metabolic processes within the body unrelated to BAT-associated heat dissipation may influence the relationship observed between the trialed interventions and the measured energy expenditure. Many studies use a low Respiratory Quotient (RQ) as a surrogate for BAT activity. Importantly, supraclavicular skin temperature positively correlates with ^18^F-FDG uptake by BAT in young healthy lean men and is therefore used as a surrogate for BAT activity (27). Many studies show an increase in heart rate in response to the trialed interventions that may be picked up by the calorimeter and may contribute to the observed increase in EE. Additionally, there are studies that conducted their trials on lean rather obese individuals, presenting a limitation on the measured EE for those studies.

We examined studies that may show a relationship between the EE and weight loss observed that can be attributed to possible BAT activity. In the β-agonist studies, there was one study by Vosselman et al., 2012 (20) that showed detectable BAT activity. In the cold exposure studies, both Yoneshiro et al., 2011 (48) and Yoneshiro et al., 2013 (51) showed increases in EE and CIT, respectively, in BAT (+) subjects. Three studies testing both cold exposure and capsinoid treatment showed increases in EE [Yoneshiro et al., 2012 (40); Ang et al., 2017 (41)] and CIT [Yoneshiro et al., 2013 (51)] in BAT (+) subjects. One study by Heinen et al., 2018 (30) administered TRH following mild cold exposure and this resulted in an increased BAT glucose uptake, possibly associated with an increase in BAT activity. Various studies also directly measured weight loss as described above.

The interest in BAT activation and thermogenesis also encompasses the possibility of not only increasing the activity or expanding the reservoir of existing BAT, but also in transitioning WAT lacking thermogenic capacity into heat-generating adipose resembling BAT (often referred to as “browning of” or “beige” WAT). Though very limited data in humans exists, phosphodiesterase inhibitors have shown promise in promoting this process through the increased availability of cGMP. The PDE5 inhibitor, sildenafil, showed evidence of promoting WAT browning in a small study in overweight men, with both metabolic changes consistent with increasing energy expenditure and BAT activation, as well as morphologic changes in WAT reflecting “browning” (31). Even with the induction of thermogenic changes in WAT, no significant change in weight was observed. Further investigation is needed given the short time frame of the study that may have limited observable changes in weight with sildenafil treatment (31).

An interest in caffeine/green tea and their ability to increase EE has also been reported, though interindividual and seasonal variations of BAT activity may be important (66). Subjects with known activation of BAT, confirmed with FDG uptake, were more likely to experience changes in EE compared to individuals with minimal BAT activity with a single dose of catechin (green tea). Daily ingestion also led to a significant increase in cold induced thermogenesis (66). These presumed positive changes in metabolism may, however, have minimal effects on weight loss or body-weight maintenance (65).

The impact of newer medications for obesity [GLP-1 receptor analogs (GLP-1 RA] on EE appears to be non-clinically significant with an effect mainly mediated by decreased energy intake (71–75). The novel GIP-GLP-1 dual agonist, tirzepatide, recently approved for diabetes management and with incredibly potent weight loss effects, may have some effect on increasing EE, however, the effect (similar to GLP-1 RA) appears to be largely mediated by reduced calory intake (76).

In conclusion, the activation of BAT remains an interesting area of investigation and possibly underestimated target in metabolic health (67). The changes observed in EE do not correlate with major weight changes with different interventions commonly known to stimulate BAT activation. Variations in the duration of interventions and the metrics utilized to estimate thermogenesis/EE make the comparisons of agents difficult to interpret. The potential side effects with β-agonists (tachycardia), sibutramine (hypertension, tachycardia), thyroid hormone (osteopenia, arrhythmias) make these agents less attractive as long-term solutions for patients with obesity. Even though cold exposure appears to consistently activate BAT and induce thermogenesis, studies are small, and it appears to be an unlikely sustainable therapy to combat obesity. Given that weight loss is associated with a decrease in REE that may favor weight regain (77) it may be ideal to identify if chronic exposure to medications that increase REE can maintain weight lost over time in people with obesity and diabetes.

Limitations of this systematic review include the potential selective publication of positive studies. A large proportion of studies were of short duration. Although we aimed to provide a comprehensive review of recent literature, we may have excluded potentially relevant studies published before the year 2000.

It is important to note that only a minority of the studies included in this systematic review directly measure BAT activity, and therefore it is difficult to assess what the contribution of BAT itself is to the change in EE. Future emphasis should be placed on determining which individuals are BAT (+) or BAT (-) for the β-agonists interventions prior to the study protocol in order to better determine if and how these interventions specifically target BAT thermogenic capacity. Future emphasis should be placed on limiting surrogates for BAT activity, such as RQ and supraclavicular skin temperature. Future studies may consider examining obese subjects where the magnitude of the effect of interventions may be larger. Future studies would benefit of longer follow up to determine the clinical relevance of influencing EE on weight loss. Finally, understanding variations in metabolism across the lifespan [i.e. progressive decline with age (78)] with their compensatory changes in energy intake may help individualize the selection of therapies that modulate EE.

## Data availability statement

The original contributions presented in the study are included in the article/**Supplementary Material**. Further inquiries can be directed to the corresponding author.

## Author contributions

FP and LCP designed the study. LCP, LTP, and YN conducted data extraction, organized the data and wrote the first draft of manuscript. GU, GD, and FP critically reviewed the manuscript. All authors contributed to the article and approved the submitted version.
