# Supplementary material for: Interventions associated with brown adipose tissue activation and the impact on energy expenditure and weight loss: A systematic review
Source: Front Endocrinol (Lausanne). 2022 Dec 9;13:1037458. doi: 10.3389/fendo.2022.1037458 (PMC9780295; doi:10.3389/fendo.2022.1037458)
Supplement: Supplementary file 1 [file Table_1.docx]

**Supplementary Table 1: Details of clinical trials investigating effect of Cold Exposure and Capsinoids on Energy Expenditure/BAT activity:**

| **Author** | **Agent** | **Study**  **Design** | **Dose** | **Population** | **Duration** | **Measurements of EE** | **Effect** | **Key Findings** |
| --- | --- | --- | --- | --- | --- | --- | --- | --- |
| **Wijers et al. (2007)** | Cold & high calorie meal were compared. | *Single arm intervention.* | 22°C and 16°C | 13 healthy male Caucasian. Mean age 22.77 yrs. and mean BMI 22.96 kg/m^2^ | 3 days. 3x respiration chamber; 2x for 36 hrs at 22°C & 1x for 84 hrs at 16°C. | EE (mJ/day) measured from VO2, VCO2, and urine nitrogen excretion. | ↑ | Mild cold exposure: significant increase in EE by 0.59 MJ/d (P < 0.001). Overfeeding: Increase EE by 0.71 MJ/d (P < 0.001). Decrease Tsk (surrogate of EE) with cold; no change Tc (surrogate of EE) with cold. |
| **Saito et al. (2009)** | Cold. | *Single arm intervention.* | 28°C and 19°C | 56 volunteers. Mean age males (35.8 ± 9 yrs.) & females (38.8 ± 8.8 yrs.). Mean BMI males (23.8 ± 2.6 kg/m^2^) & females (21.1 ± 2.3 kg/m^2^) | 7 months. 71x and 2 hrs total/time: 1 hr under 19°C cold condition, then 1 hr FDG and 19°C cold, or 2 hrs warm conditions (with FDG uptake). | FDG-PET/CT performed after 2nd hr of exposure to cold. Measurement of EE not specified. | ↑ | FDG uptake (surrogate of EE) in supraclavicular & paraspinal regions of adipose tissue. BAT 1/∞ age. FDG uptake 1/∞ BMI, total fat, and visceral fat; pointing to greater BAT activity in lean subjects. |
| **Van Marken et al. (2009)** | Cold. | *Single arm intervention.* | 22°C and 16°C | 24 men. 10 lean (BMI 23.2 ± 3.6 kg/m^2^), 14 obese (BMI 30.3 ± 4.2 kg/m^2^). Mean age lean (24.3 ± 3.6 yrs.) and obese (23.5 ± 3.4 yrs.) | 1 day: 9AM-1PM after an overnight fast beginning at 10 PM the night before. 1 hr. for 22°C & 2 hrs. for 16°C. | EE measured by a respiratory gas analyzer with the use of a ventilated hood system. FDG-PET/CT performed after 2nd hr of cold exposure. | ↑ | Significant increase in Tc: 0.13 ± 0.16°C in lean and by 0.16 ± 0.22°C in obese (P < 0.005) Significant drop in Tsk in both groups: 3.4 ± 0.9°C in lean and by 3.7 ± 0.5°C in obese (P < 0.005). BAT activity highest in the supraclavicular region. BAT 1/∞ age. BAT activity 1/∞ BMI and body fat |
| **Snitker et al. (2009)** | Capsinoid. | *Double-blind randomized, placebo- controlled trial.* | 6 mg/d of capsinoids orally, or placebo. | 40 men/40 women. Mean age of 42 ± 8 yrs. and BMI of 30.4 ± 2.4 kg/m^2^ | 5 visits over 13 wks. | REE (kcal/d) only measured in men through indirect calorimetry. Body weight (kg) through X-ray absorptiometry. | ↑ | REE was 54 kcal/d higher in capsinoid group than in placebo. Weight change at 12 wks. was 0.92 ± 3.12 kg in capsinoid group and -0.49 ± 2.37 kg in the placebo group. |
| **Celi et al. (2010)** | Cold. | *Randomized, single-blind, and cross-over intervention trial.* | 24°C and 19°C. | 25 volunteers; 15 male/10 female. Mean age 28.6 ±8.5 yrs. Mean BMI 23.3 ± 2.2 kg/m^2^ | 2-day diet, then assignment into 19°C or 24°C for 12 hrs. Then, 36 hr recovery followed by 12 hrs at the other study temp. | EE (kcal/h) measured by use of a whole room indirect calorimeter. | ↑ | At 19°C, EE increased in 18 of 21 subjects. 12 hr EE at 24°C was 82.09 ± 8.61 kcal/hr and at 19°C was 86.98 ± 9.93 kcal/hr (P < 0.001). Increase in EE both pre- and postprandially; greater at 19°C (P < 0.001). Decreased Tsk and no change Tc (P < 0.001). |
| **Wijers et al. (2010)** | Cold. | *Single arm intervention.* | 22°C and 16°C | 10 groups of 10 male subjects: lean group mean BMI of 22.6 kg/m^2^ and obese group mean BMI of 33.5 kg/m^2^. Lean mean age (23 yrs.) and obese (29 yrs). | 84 hr. respiratory chamber: 36 hrs. at 22°C followed by 48 hrs. at 16°C. | EE (MJ/day) was determined from subjects’ O2 consumption, CO2 production, urine nitrogen excretion. TDEE calculated by multiplying SMR x physical activity index of 1.55. | ↑ | EE significantly higher in obese subjects than in lean, both in baseline condition (12.92 and 11.35 MJ/day, respectively, p < 0.01) and after mild cold (12.97 and 11.60 MJ/day, respectively P < 0.01); however, with a greater difference in EE between lean from baseline to cold. Decrease Tsk obese & Increase Tc obese. EE 1/∞ Tsk. |
| **Josse et al. (2010)** | Capsinoid | *Double-blind, randomized, placebo-controlled trial.* | 10 mg capsinoids at rest, or placebo | 12 healthy young men of mean age 24.3 ± 3 yrs. and mean BMI of 25.5 ± 1.7 kg/m^2^ | Duration not specified. | All data were analyzed using a repeated measure two-way ANOVA Analyzed using Tukey’s test. Plasma NE analyzed with HPLC. | ↑ | Increase in REE by approximately 20%. Fat oxidation at rest (placebo): 1.18 kcal/min to 1.26 kcal/min and with capsinoid: 1.19 kcal/min to 1.41 kcal/min. Decrease in visceral fat. Increase plasma NE. |
| **Yoneshiro et al. (2011)** | Cold. | *Single arm intervention.* | 27°C and 19°C | 13 male volunteers. Mean age BAT (+) 22.7 yrs. and BAT (-) 22.9 yrs. Mean BMI BAT (+) 20 kg/m^2^ and BAT (-) 21.4 kg/m^2^ | Fasting 6-12 hrs., followed by 2 hrs. in 19°C. | EE (kcal/day) measured by respiratory gas analyzer connected to a ventilated hood. FDG-PET/CT performed after 2nd hr. of exposure to cold at a temp of 24°C. | ↑ | 27°C: EE was 1,446 ± 97 kcal/day in BAT (+) group, not differing from BAT (-). 19°C: EE increased to 1,856 ± 218 kcal/ day in BAT (+) group (p < 0.05); rose slightly to 1,475 ± 206 kcal/day in BAT (-) group. 19°C: significant drop (P < 0.01) in Tsk of supra-clavicular region of BAT (-) group; small drop in BAT (+). |
| **Ouellet et al. (2012)** | Cold. | *Single arm intervention.* | Room temp (22C-25C) and 18°C. | 6 healthy men. Age range 23-42 yrs. BMI range of 23.7 to 31.0 kg/m^2^ | 300 min: 0-120 min at room temp and 120-300 min at a cold exposure temp of 18°C. | EE measured by indirect hood calorimetry. FDG-PET/CT performed upon cold exposure. C-acetate kinetics to determine tissue oxidative capacity. | ↑ | REE, VO2, and VCO2 increased approximately 1.8-fold Tsk reduced by 3.8 ± 0.4°C without change in Tc. |
| **Yoneshiro et al. (2012)** | Capsinoids and Cold. | *Single-blind, randomized, placebo-controlled crossover trial.* | 9 mg of capsinoid with 100 mL of water in 1 min, or placebo. | 18 healthy men aged 20-32 yrs. and who lived in Sapporo for > 3 yrs. Mean BMI not specified. | 2 hrs. of cold (19℃) after oral ingestion of capsinoid  2 hours under warm conditions (27℃). 4 wks later, 2 tests on response of the body conducted 1-3 wks apart. | Measurement of EE not specified. | ↑ | Cold acclimation before capsinoid ingestion. 10 BAT (+) subjects showed FDG uptake. EE increased by 15.2 ± 2.6 kJ/d in 1 hour for BAT (+) and by 1.7 ± 3.8 kJ/d for the BAT (-) after oral capsinoid ingestion (P < 0.01). No change Tsk. |
| **Chen et al. (2013)** | Cold. | *Randomized, single-blind, and cross-over intervention trial.* | 24°C and 19°C | 24 volunteers: 14 males/10 females. Age range 19-60 yrs. BMI of 20.0 –27.0 kg/m^2^ | 2-day equilibrium diet followed by 12 hrs. in 19°C or 24°C conditions. After 36 hrs., crossing over to alternate study temp. | EE measured by a whole room indirect calorimeter (respiration chamber). FDG-PET/CT performed after 1 hr in respiration chamber. Study design allowed attributing changes in EE to CIT response. | ↑ | Increase in EE (5.3% ± 5.9%, P < 0.001) greater in women than men (9.1% ± 4.3% vs 2.3% ± 5.3%, p < 0.002). 7 subjects showed 18F-FDG uptake at 19°C. 19°C: mean SUV, BAT volume, & maximum SUV torso-mantle increased 10.5% (P < 0.001). EE ∝ SUV, EE 1/∝ age, and BAT activity 1/∝ NE levels. Significant drop in Tsk. FDG uptake highest in cervical, supra-clavicular, thoracic regions. |
| **Schlogl et al (2013)** | Cold & high calorie meal. | *Proof-of-concept study.* | 22°C and 16°C | 16 healthy adults. Mean age 30.9 ± 9.9 yrs. Mean BMI 26.4 ± 5.5 kg/m^2^ | Proof of concept: 6 subjects overnight fast & 2 hrs. at 16°C, then first 6 subjects at 36 hrs. fasting & 22°C. Overfeeding: 10 subjects 2 hrs. at 16°C, then all subjects 24 hrs. overfeeding. | FDG-PET/CT after exposure to 16°C and 22°C; as well as, after the 24-hr. overfeeding. CIBA (cold induced BAT activation). Measurement of EE not specified. | ↓ | Proof of concept: CIBA in 4/6 subjects; Decrease 24 hr EE by -8.3% ± 2.7% (P = 0.001); Overfeeding: CIBA in 8/10 subjects. Increase 24 hr EE by 7.5% ± 5.7% (P = 0.008) with overfeeding subjects. Increase in EE despite no BAT activation. |
| **Van der lans et al. (2013)** | Cold. | *Single arm intervention.* | 15°C -16°C | 17 healthy subjects: 9 female, 8 males. Mean age 23 ± 3.2 yrs. And Mean BMI 21.6 ± 2.2 kg/m^2^ | 15°C–16°C for 10 consecutive days: 2 hours on the first day, 4 hours on the second day, and 6 hours per day for the remaining days. | EE measured via indirect hood calorimetry. | ↑ | Significant (P < 0.05) Increase in EE: 6.2 ± 0.7 to 6.9 ± 1.0 MJ/24 hrs. (females) and 7.6 ± 0.7 to 8.5 ± 0.6 MJ/24 hrs. (males). Significant increase NST (P < 0.05): 10.8 ± 7.5% to 17.8 ± 11.1%. Increased upper body BAT activity from 2.4 ± 0.7 to 2.8 ± 0.5 SUV mean (P < 0.01) and detectable BAT volume from 665 ± 451 cc before to 913 ± 458 cc after (P < 0.01). Decreased Tsk. |
| **Yoneshiro et al. (2013)** | Cold and capsinoids (non-pungent capsaicin analogs) - chemical stimulants of TPR channels. | *Single arm intervention.* | 27°C, 19°C, and 17°C. 9 mg capsinoids. | 51 healthy male volunteers: Mean age, 24.4 ± 0.5 yrs. And mean BMI 22.0 ± 0.4 kg/m2. | Subjects exposed to 27°C followed by 2 hrs. at 19C. Afterwards, 1/22 subjects exposed to cold at 17°C for 2 hours every day for 6 weeks. 10 subjects (BAT -) capsinoid ingestion 1/day for 6 wks.; with cold before & after treatment. | EE measured using a respiratory gas analyzer. FDG-PET/CT after 2 hrs. of cold exposure at 19°C. CIT measured by difference between EE at 27°C and 19°C. | ↑ | Cold acclimation before capsinoid ingestion. EE increased in BAT (+) and (-) subjects. CIT significantly higher in BAT (+) than BAT (-) subjects (252.0 ± 41.1 vs 78.4 ± 23.8 kcal/d, P < 0.01). In individuals with low or undetectable BAT activity, the CIT after capsinoid treatment (200.0 ± 33.9 kcal/d) higher than before capsinoid treatment (20.6 ± 43.0 kcal/d, P < 0.01). 2% reduction in body fat. |
| **Blondin et al. (2014)** | Cold. | *Randomized controlled trial.* | 25°C, 18°C, and 10°C | 6 healthy/lean men. Mean age 23 ± 1 yr. Mean BMI of 24.5 ± 1.2 kg/m^2^ | 2 hr. cold acclimation (10°C), repeated 5 consecutive days/wk. for 4 consecutive wks. 120 min baseline period at 25°C followed by 180 min of exposure to cold (18°C). | Whole-body metabolic heat production was determined by indirect respiratory calorimetry; EE (kcal/min). | ↑/↓ | EE before cold acclimation: 25°C (1.4 ± 0.1 kcal/min); cold (2.7 ± 0.2 kcal/min) (P < 0.05). EE after cold acclimation: 25°C (1.3 kcal/min); cold (2.5 ± 0.2 kcal/min) (P < 0.05). BAT activity increased 45% after 4 wks of cold: 66 ± 30 mL before acclimation vs 95 ± 28 mL after acclimation (p = 0.05). |
| **Chondronikola et al. (2016)** | Cold. | *Single arm intervention.* | 23°C-24°C and 19°C-20°C | 18 men. BAT (-)/ 10 BAT (+) men. BAT (+) age (38.4 ± 15.3 yrs.); BMI (28.2 ± 5.4 kg/m^2^). BAT (-) age (57.5± 16.2 yrs.); BMI (31 ± 3.2 kg/m^2^). | Overnight rest: 23°C-24°C. Morning: 6 hrs. & individualized cold. Temp of room lowered by 1°C every 30 min until shivering. Then, temp increased by 1°C to prevent shivering. | FDG administered at 5 hr. after cold exposure; 1 hr later - PET/CT. Individualized cold exposure protocol was employed to maximally induce NST. Measurement of EE not specified. | ↑ | BAT (+): higher BAT metabolic activity vs BAT (-) subjects (1 g/ml SUV compared to 2.2 g/ml SUV, P < 0.05). Tc decreased in the BAT (-) group; increased in BAT (+) group. Significant decrease in Tsk at supra-clavicular region in BAT (-) group (-2°C in BAT (-) vs 0.5°C in BAT (+)). |
| **Haman et al. (2016)** | Cold. | *Single arm intervention.* | 7.5°C | 8 healthy men. Age range 22-47 yrs. Mean BMI 28.5 kg/m^2^. | 24 hrs. 12 hr. fast followed by 7.5°C for 12-24 hrs. (6 subjects completed 12 hrs. & 2 completed the 24 hrs.). | EE measured by indirect hood calorimetry. | ↑ | Tsk decreased from 32.8°C to 28.1°C after 12 hrs. Tc: unchanged for first 6 h, then decreased from 37.4°C to 36.8°C after 12 hrs. By the end of 24 h, the median Tc was 36.5°C; not significantly different from baseline. |
| **Hanssen et al. (2016)** | Cold. | *Single arm intervention.* | 14°C – 15°C | 10 overweight/obese males. Mean age 36 ± 13 yrs. Mean BMI 32.9 ± 3.5 kg/m^2^ | 10 days. 2 hrs. on 1st day, 4 hrs. on the 2nd day, and 6 hrs. on the 3rd through the 10th days. | FDG-PET/CT taken before and after cold exposure. Individualized cooling protocol. NST measured as increase in EE to mild cold above BMR. Measurement of EE not specified. | ↑ | Acute cold: significant increase in EE before cold acclimation (5.9 ± 0.8 to 6.5 ± 0.8 kJ/min (P < 0.01) and after cold acclimation (5.7 ± 0.7 to 6.5 ± 0.8 kJ/min (P < 0.01) cold. Increased mean BAT SUV from 2.3 ± 0.3 to 2.9 ± 0.3 (P < 0.01) and maximal BAT SUV from 8.8 ± 3.7 to 18.9 ± 5.5 (P <0.01). |
| **U. Din M et al. (2016)** | Cold. | *Single arm intervention.* | 6°C and increasing in increments. Room temp: 22°C. | 7 healthy male and female subjects. Mean age 36 ± 11 yrs. Mean BMI 25.5 ± 3.3 kg/m^2^ | Cold started 2 hrs. prior to scan with cooling blankets; cooling blanket temp at 6°C. Temp was gradually raised once subjects were observed to be shivering or reported shivering. | EE measured via indirect hood calorimetry. FDG-PET/CT at room temp and with cold. | ↑ | EE significantly higher after cold as compared with room temp condition (1701 ± 282 versus 2052 ± 574 kcal/24 hrs respectively, P = 0.046). Increase in whole-body VO2 during cold stimulus (room temp: 248.3 ± 40.2 versus cold: 299.6 ± 82.3 mL/min, P = 0.04). |
| **Peterson et al. (2016)** | Cold & high calorie diet. | *Single arm intervention.* | 4°C, 16°C, & 22°C | 9 healthy, lean men. BMI 18.5–25 kg/m^2^. Age range 18-35 yrs. | 20 mins/day, 5 consecutive days/wk, for 4 wks. (4°C; acute cold). Then, a 2-day testing protocol for EE at both 22°C and cold (16°C). Then, resp. chamber for 24 hrs. and overfeeding diet at 22°C. | EE was measured by indirect calorimetry using a ventilated hood system. Overfeeding diet at 50% above energy requirements. CIT calculated as percent increase in RMR from thermoneutral to cold. | ↑ | After 4 wks. of acute cold: avg. loss of 0.5 ± 0.5 kg or 1.1 lbs (P > 0.05) 16°C: RMR increased from 1782 ± 198 kcal/24 hrs during baseline to 1824 ± 214 kcal/24 hrs post-intervention testing. CIT was 5.2 ± 14.2% at baseline and increased to 12.0 ± 10.6% after 20 sessions of cold exposure (P = 0.05). |
| **Romu et al. (2016)** | Cold. | *Randomized controlled trial.* | 21°C, or no cold exposure. | 28 total subjects; 16 cold/12 control. Mean age cold 26.1 ± 3.9 years. Mean BMI cold 21.6 ± 1.2 kg/m^2. | 18-28 min duration. Registration: underwear and blanket at 21°C. After 8–14 min, subjects drank a glass of ice-cold water (3 dl) and a cold vest. | A ventilated hood technique used to measure metabolic rate, as based on O2 inhalation and CO2 exhalation. | ↑ | Non-significant increase of metabolic rate between baseline and cold. Increased BAT volume. Study took place in Sweden; therefore, some adaptation to cold may have occurred. Increase in body weight of control subjects only. |
| **Ang et al. (2017)** | Capsinoid capsule or Placebo and cold (18 ℃) | *Prospective double blind, randomized, placebo- controlled trial.* | 1.5 mg in each capsule (9mg in total) -6 capsules consumed, or placebo. | 24 healthy, lean male volunteers (BMI <25 kg/m^2) - High BAT group (6), Low BAT group (18). | Duration not specified. | EE and RQ measured by indirect calorimetry. BAT activity in the supra-clavicular region measured using IRT video imaging. | ↑ | Cold acclimation before capsinoid ingestion. Capsinoids: increase EE & fat oxidation. BAT (+) group: increase in EE higher in capsinoid group. (P = 0.01). BAT (+) group showed higher increases in fat oxidation following capsinoid consumption compared to placebo (P = 0.03). After ingestion of capsinoid, BAT (+) group showed higher heat output (P = 0.01). |
| **Acosta et al. (2018)** | Cold. | *Study design not specified.* | 22°C-33°C and 19.5°C-20°C | 11 Caucasian male adults. Mean Age: 23 ± 2 years; Mean BMI: 23.1 ± 1.2 kg/m^2^ | 55 mins: 20 min pre-start at 22°C-23°C, then 20 min in 23°C-33°C. Then, 5 min at 19.5°C-20°C followed by wearing a vest at 16°C to 1.4°C until shivering. | EE measured by indirect hood calorimetry. | ↑ | Mean total increase in REE was 31.7%. EE increased from 1600 - 1800 kcal/day from warm to 31% of cold exposure; decreased from 1800 - 1700 kcal/day from 31% to 64% of cold exposure; & increased from 1700 - 2000 kcal/day from 64% of cold exposure to the shivering threshold. Decrease in Tsk. |
| **Loh et al. (2018)** | Cold & Pioglitazone (TZD). | *Blinded, placebo controlled, parallel trial.* | 45 mg/day with a cold intervention of 14°C & lowering by 1°C every 5 mins until shivering, or placebo | 14 male participants (14 women & 12 men). Age range 19–30 years, BMI ≤ 25 kg/m2. | 28 days. | Whole body EE measured by indirect calorimetry. Standardized cold exposure via a temperature controlled, water-perfused vest and blankets. | ↔ | Basal and cold-stimulated energy expenditure and respiratory exchange ratio (RER) remained unchanged after chronic pioglitazone treatment. Decreased SUV with Pioglitazone; decrease in cold induced glucose uptake. Reduced BAT activity. Weight gain. |
| **Senn et al. (2018)** | Cold. | *Data collected from a prospective observation study and an intervention trial.* | 25°C-10°C | 56 subjects. Mean age and BMI not specified. | 2 wks. Subjects in hospital bed & covered with a fleece blanket. Then, remove blanket & clothes. After, exposed to cold.  Water temp reduced by 1°C every 2 min from 25°C to a minimum of 10°C. | Energy expenditure (kcal/day) was measured by indirect calorimetry for 30 min. Cold stimulus using a water circulated cooling system. CIT defined as difference between EE cold and EE warm. | ↑ | Outdoor temperatures significantly correlated with EE in cold condition. CIT 1/∝ outdoor temperature; pointing to the importance of cold acclimation. |
| **Sun et al. (2018)** | Capsinoids and cold | *Crossover design.* | 12 mg of Capsinoids or ≤ 2 hrs. cold exposure (~14.5 ℃) | 20 healthy participants (8 men, 12 women) aged 21-35 yrs. and a BMI of 18.5-26.0 kg/m^2. | 4 experimental trials separated by a minimum 48 hrs. | EE fat oxidation, respiratory quotient measurements. BAT activation visualized by FDG-PET/CT analysis. | ↑ | EE increased significantly in BAT (+) group: 146 kcal/2 h after capsinoids, 300 kcal/2 h after cold. Capsinoids stimulate BAT to a lesser degree than cold. Capsinoid elicit no detectable BAT stimulation. BAT SUV of 2.9 and avg. BAT volume 75 cm after cold. |

EE (energy expenditure), TDEE (total daily energy expenditure), WL (weight loss), CIT (cold induced thermogenesis), NST (non-shivering thermogenesis), DIT (diet-induced thermogenesis), CIBA (cold-induced BAT activation), Tsk (skin temperature), Tc (body core temperature), Tb (mean body temperature), VO2 (oxygen consumption), RER (respiratory exchange ratio), RMR (resting metabolic rate), SMR (standard metabolic rate), BAT (brown adipose tissue), WAT (white adipose tissue), SUV (standard uptake value), SNS (central nervous system), FFA (free fatty acid), NEFA (non-esterified fatty acid), NE (norepinephrine), TSH (thyroid stimulating hormone), UCP (uncoupling protein), 1/∝ (inverse relationship), ∝ (direct relationship).
